# ‘Better Conversations With Developmental Language Disorder’: Designing a Novel Intervention for School‐Aged Children and Their Main Carers

**DOI:** 10.1111/1460-6984.70234

**Published:** 2026-04-15

**Authors:** Lucy Hughes, Liz Croot, Caroline Newton, Wendy Best

**Affiliations:** ^1^ Moor House Research & Training Institute Moor House School & College Oxted UK; ^2^ School of Psychology and Clinical Language Sciences University of Reading Reading UK; ^3^ Department of Language and Cognition University College London London UK; ^4^ School of Medicine and Population Health University of Sheffield Sheffield UK

**Keywords:** children, conversation, developmental language disorder, intervention development, parent‐child interaction

## Abstract

**Background:**

Children with developmental language disorder (DLD) experience difficulties with receptive and expressive language, which can affect their participation in everyday interactions with others. This, in turn, can impact their ongoing language development and ability to form and maintain friendships. This paper describes the development of a complex intervention, ‘Better Conversations with Developmental Language Disorder’ (BCDLD), designed with and for school‐aged children with DLD and their main carers.

**Methods:**

Medical Research Council guidelines for developing complex interventions were used to inform the design and early‐stage evaluation of BCDLD, including consultation with key interest holders at each stage of the project; appraisal of relevant literature and articulation of programme theory.

**Results:**

A review of the evidence for communication partner training revealed a gap in the research and clinical practice for school‐aged children with DLD. Research with other clinical groups suggests potential for enhancing everyday interactions through work with conversation partners. Theory underpinning the proposed intervention, which was identified from the review, included social interactionist and constructivist accounts of child language acquisition, behaviour change theory and applied conversation analysis. Children and parents with experience of living with DLD identified barriers to their everyday conversation to inform intervention content and delivery. Speech and Language Therapists gave feedback on the draft intervention protocol and advised on how BCDLD could be implemented in clinical practice.

**Conclusion:**

This paper reports the development of a theoretically‐informed, conversation‐based intervention for children with DLD and their conversation partners, which aims to improve the content and flow of their everyday interactions, whilst supporting children's language development. Synthesising knowledge from the literature, input from children living with DLD, parents and professionals in the field enabled the design and early‐stage evaluation of BCDLD.

**WHAT THIS PAPER ADDS:**

*What is already known about this subject*

- There is a gap in the literature and clinical practice for a conversation‐based intervention, designed for school‐aged children with DLD and their parents or carers.

*What this paper adds to existing knowledge*

- This project followed Medical Research Council guidelines for early development of a novel intervention, ‘Better Conversations with Developmental Language Disorder’.

*What are the potential or actual clinical implications of this work?*

- The programme is ready to progress to the next stage of development, including manualisation and training, leading to feasibility studies within NHS and education settings.

## 1 Introduction

Developmental language disorder (DLD) affects 1 in 14 children at school entry (Norbury et al. 2016) and is associated with poor educational and mental health outcomes (Ziegenfusz et al. 2022; Lim and Lum 2024). Children with DLD experience difficulties with receptive and expressive language, which can affect their participation in everyday interactions with family and peers. This, in turn, can impact their ongoing language development and their ability to establish and maintain social relationships. Research Priority Setting Partnerships have identified a need for interventions that address communication and functional outcomes, individualised goals and self‐help strategies for people with DLD (Kulkarni et al. 2022; Royal College of Speech and Language Therapists 2025). Yet, there is a lack of evidence‐based programmes which target the use of language in real life contexts, such as natural conversation, tailored to individuals within this clinical group.

Communication partner training interventions, including those described elsewhere within this Special Issue, offer a well‐established speech and language therapy (SLT) approach, which is used widely with other clinical populations, for example, stroke‐related aphasia (Best et al. 2016), language‐led dementia (Volkmer et al. 2023) and Parkinson's (Bloch and Beeke 2022). These programmes offer a practical approach, focused on identifying strategies for both the client and partner to support their everyday conversation. Outcomes from adults with acquired communication disorders suggest that a ‘Better Conversations’ approach (Beeke and Bloch 2022) may have potential to improve the content and flow of dyadic conversations and increase communicative participation. However, there is a need to adapt existing protocols for the specific needs and circumstances of children with DLD.

Intervention development entails the bringing together of existing evidence and theory, combined with the views of key interest holders and consideration of the context in which the programme will be delivered (Skivington et al. 2021). This process typically follows four phases, as outlined by the Medical Research Council (Craig et al. 2006). Phase I focuses on the initial planning and design of a therapy protocol, which is then reviewed and refined before proceeding to wider feasibility testing (Phase 2), evaluation (Phase 3) and implementation (Phase 4). This paper describes Phase 1, the early stage development of a complex intervention, ‘Better Conversations with Developmental Language Disorder’ (BCDLD), which aims to meet the needs of school‐aged children with DLD and their main carers. O'Cathain et al. 2019 offer a framework for Phase I intervention development, which includes the actions summarised in Table 1.

This framework supports theory‐driven intervention development and stresses the importance of delivery context, while building on the existing evidence base. These authors emphasise that the process of following core actions in the development of an intervention may not be linear and may involve cycling back through earlier stages to ensure that the intervention is acceptable, feasible and desirable to its intended beneficiaries, while avoiding unintended harm. Therefore, the actions proposed by O'Cathain et al. (2019) are described and presented within this paper as forming part of Phase I intervention development, which followed three interlinked sub‐stages for BCDLD. In Stage 1, we report on how stakeholder involvement and clinical expertise informed the creation and refinement of this novel intervention. Stage 2 focuses on the review of literature, which provided a theoretical and evidence‐based grounding for the BCDLD programme. Finally, Stage 3 describes the design of a logic model and therapy protocol to guide intervention delivery by trained and qualified clinicians. A ‘GUIDance for rEporting intervention Development studies in health research’ checklist (GUIDED; Duncan et al. 2020) is provided in Supplementary Materials 1 to further support the comprehensive description of the process and ensure completeness of reporting.

**TABLE 1:** Summary of principles and actions to inform Phase 1 intervention development, based on O"Cathain et al. 2019).

Key principles	Actions
Dynamic	Plan the development process
	Involve interest holders, including those who will deliver, use and benefit from the intervention
Iterative	Bring together a team and establish decision‐making processes
	Review published research evidence
Creative	Draw on existing theories
	Articulate programme theory
Open to change	Undertake primary data collection Use a wide range of research methods throughout, for example, qualitative research to understand the intermediate outcomes
	Understand context
Looking towards evaluation	Pay attention to future implementation of the intervention in the real world
	Design and refine the intervention

## 2 Stage 1: Drawing on Interest Holder Involvement and Clinical Guidance to Establish a Need for the Intervention, Understanding and Specifying the Delivery Context

This stage drew on interest holder input and clinical information from different sources, including: (a) school‐aged children with DLD, their parents and teachers; (b) specialist clinicians, researchers and a clinical linguist, all with extensive experience in working with this client group; (c) Royal College of Speech and Language Therapists (RCSLT) clinical guidance and consultation on priorities for DLD; (d) an initial review of literature to define the intervention context and target group.

### 2.1 Initial Consultation With Interest Holders

This began while the first and final authors were working together on the Word Retrieval and Development Project (WoRD; Best et al. 2021), an intervention study focusing on lexical therapy for children with DLD. Twenty children with DLD participated in the study, which was approved by UCL Ethics Committee (reference number 281/002). As part of the project, child participants, their parents and teachers were invited to share their experience of living with DLD and their communication support needs via structured interviews (children) and questionnaires (adults). A content analysis of this data was carried out by a student researcher, who was independent of the core project team, in line with Hilari et al. (2023) and Palmer et al. (2017). The researcher grouped topics which were raised frequently by participants and highlighted key quotes to illustrate these topics. First, children identified barriers to their everyday conversation, which affected their education, friendships and family relationships. One child (aged 7 years) expressed her frustration at not being able to get her message across at home and at school. She commented:

‘It can be quite annoying at home, because my sister speaks over me. At school, I get really angry, because I forget and can't tell the answer… People say things instead of me.’

Another child (aged 8 years) described how her DLD affects her participation in conversations with friends:

‘Talking to my friends and I didn't say a word—I didn't even know what to say’.

A third child (aged 7 years) described what conversation meant to her:

‘If we can't have conversation, then people won't be friends’.

These insights from children were supplemented by feedback from parents and teachers, who collectively identified word‐finding difficulties and going off topic as the main barriers to conversation. A topic which arose frequently within parent responses was a need for more advice and strategies to help them to support their children's communication at home. One stated:

'Sometimes, he can't explain what's happened or what's wrong, so he gets angry or cross. I don't know the best way to understand him or to help'.

Another reported:

‘He does a lot of “um um um” and then starts back at the beginning and then says “um um um” again. I tell him to stop, think, and start again slowly. I think he gets anxious because he knows he's not very good at communicating'.

Finally, teachers highlighted the effects of a child's language difficulties in school, one teacher said:

‘She finds social situations difficult as she struggles to express herself’.

Another observed:

‘It limits her understanding of instructions and can affect her ability to socialise’.

A third teacher commented:

‘I think sometimes he can do it but is afraid to have a go in front of the class in case he gets it wrong, and in case he gets teased… He doesn't like to be picked in front of a group’.

These comments from children and adults who took part in the WoRD study motivated the focus on everyday conversation when developing BCDLD, because of its perceived impact on social participation and access to learning.

### 2.2 Input From Clinical Advisory Group

Following this initial feedback and consultation with people living with DLD, a Clinical Advisory Group was set up, including all co‐authors, two specialist DLD clinicians and a senior SLT researcher, who was involved in developing the ‘Better Conversations with Aphasia’ programme (BCA; Best et al. 2016). The paediatric SLTs within the Clinical Advisory Group confirmed that children on their caseloads commonly presented with difficulties in conversation. They cited a lack of available interventions to address these difficulties, beyond social skills programmes (e.g. Kelly 2024; Rinaldi 2024), which are typically aimed at autistic children and/or those with social, emotional or behavioural difficulties. One clinician, who was working in mainstream schools, also alluded to difficulties engaging parents in therapy, whilst acknowledging that this would be best practice to support children and help generalise targets to their everyday communication.

### 2.3 Clinical Guidance

The RCSLT's Clinical Guidance for therapists working with DLD (Taylor‐Goh 2017) helped underpin the intervention's development by providing key principles, which were prioritised when making decisions during the intervention development process. This guidance states that SLT intervention aims to:

- Develop the language abilities of children with developmental language disorder to their maximum potential.
- Teach strategies to the child and those around the child to reduce the impact of their difficulties on communication and their access to education and social activities.

In addition, the guidance defines the role of SLTs as including:

- Supporting parents and
- Facilitating communication in functional settings.

Following these principles and the above interest holder input, we planned intervention which involved both children and their parents or carers, focusing on developing child language and conversation by teaching supportive communication strategies, which could be used within their everyday interactions at home.

Development took place during a period of consultation by the RCSLT with clinicians, patients and carers to identify priorities for new DLD research. This ongoing consultation was taken into account when determining the focus for intervention. The Priority Setting Partnership (Kulkarni et al. 2022), identified a Top 10 list of DLD research priority areas, which aligns with the individual views reported above. These priorities include:

- Specific characteristics of evidence‐based DLD interventions which facilitate progress towards the goals of an individual with DLD.
- Effective interventions targeting receptive language for individuals with DLD.
- Effective ways of teaching self‐help strategies to children and young people with DLD.
- Outcomes for individuals with DLD across settings (e.g., language provision, mainstream school) in relation to curriculum access, language development and social skills.

BCDLD aimed to address these priorities by offering individualised intervention, which targets both receptive and expressive language and includes the provision of self‐help strategies, aimed at increasing children's social participation and access to education.

### 2.4 Delivery Context

An initial review of the literature was conducted to further understand the clinical context with regard to existing practice and to define the target intervention group. This confirmed a lack of conversation‐focused, parent‐mediated interventions for primary‐aged children with DLD. Most speech and language interventions for children at Key Stage 1 and beyond focus on training education staff, such as teaching assistants (TA's), to support children's oral language (Roulestone et al. 2012). However, there is limited evidence for the effectiveness of these TA‐led programmes (Dimova et al. 2021; Thurston et al. 2016). Direct, clinician‐led therapy programmes aimed at school aged children may target vocabulary and word‐finding (Best et al. 2021; Ebbels et al. 2012; Wright et al. 2018), comprehension and production of grammar (Balthazar et al. 2020; Calder et al. 2021; Ebbels, 2013) or narrative structure (Gillam et al. 2014; Hessling and Schuele 2020; Spencer and Petersen 2020). Where conversation‐based programmes exist, they tend to focus on the individual child, targeting pragmatic skills such as turn‐taking and topic initiation (e.g. Adams et al. 2018; Jensen de López et al. 2022). Neither of the above studies are aimed directly at dyadic communication as it occurs in natural home or school settings.

The value of parental participation in children's speech and language therapy (SLT) is well established in the early years literature. For a review of parent‐mediated interventions, see Roberts et al. (2019). Among the most widely used therapy approaches is the Hanen ‘It Takes Two to Talk’ programme (Pepper et al. 2004), which includes group training sessions for parents to develop knowledge and understanding of language development and supportive communication strategies, as well as three home visits by a Speech and Language Therapist. A related intervention approach is parent‐child interaction therapy (PCIT; Falkus et al. 2016). Like Hanen, PCIT employs video recordings of play sessions between adults and their children to highlight key communication strategies and support parents to reflect on their own interaction style. However, the delivery of therapy differs in that PCIT typically takes place in clinic and is condensed into 4–6 individual sessions, with no additional group training.

Despite promising results from studies with pre‐schoolers, parent‐mediated interventions for school‐aged children remain an understudied area.  BCDLD aimed to address this gap directly by offering an SLT‐led, family‐focused model based on existing successful approaches in other populations (e.g., PCIT and ‘Better Conversations with Aphasia’, BCA, Best et al. 2016). It was hypothesised that caregiver involvement in treatment would be associated with positive gains for primary school pupils.

**Definition of the Target Group**. The target population of 6–8‐year‐olds with DLD was chosen based on prognosis data and developmental context. We planned to focus on this age group because language difficulties that are still evident at five years and over are likely to persist (Bishop et al. 2017). Furthermore, from Key Stage 1 (school years 1 and 2), the language demands in the classroom increase and DLD may be more clearly distinguished from other diagnoses, such as sensory or behavioural problems.  Additional inclusion criteria were defined following discussion with the Clinical Advisory Group, regarding who would be most likely to benefit from the intervention. Full inclusion criteria for the initial BCDLD intervention study (Hughes et al. 2025) are presented in Table 2.

**TABLE 2:** Inclusion criteria for BCDLD.

Inclusion criteria	Rationale
Child aged between six and eight years.	Persisting language difficulties at this age are suggestive of poor prognosis (Bishop et al. 2017).
Child presents with difficulty producing or understanding language that affects everyday functioning, as reported by parents and school and confirmed by formal language testing. Child initially referred by school SENCo; parental concern indicated by initial case history and questionnaire. Scaled score of 7 or below on two or more Clinical Evaluation of Language Fundamentals (CELF‐5; Wiig et al. 2017) core language subtests.	Bishop et al. (2017) define language disorder as ‘problems with language [which have] a significant impact on everyday social interactions or educational progress’. Bishop et al. (2016) advocate that ‘Multiple sources of information should be combined in assessment, including interview/questionnaires with parents or caregivers, direct observation of the child, and standardized age‐normed tests or criterion‐based assessments.’ This is in order to gain a full picture of the functional impact of DLD.
Having English as a main language. Exposed to English at home and/or in an English‐speaking nursery since the age of three.	These criteria for defining a ‘main language’ align with early years guidance, which identifies home language exposure and early years setting exposure as key determinants of a child's language profile (Early Years Coalition 2021). This does not exclude the child having one or more additional languages.
No other significant developmental diagnosis, which may affect the child's participation in BCDLD. For example, autism spectrum disorder (ASD), ADHD, emotional or behavioural difficulties.	BCDLD was designed for children for whom language is their primary barrier to learning and interaction. One child was excluded from the project as a result of having an additional diagnosis of ADHD and concerns from his parent and teacher that the proposed intervention may not fully meet his support needs.
Non‐verbal skills task at or above the low average range (as indexed by a percentile score ≥ 8 on the Pattern Construction task from the British Ability Scales, Third Edition (BAS‐3; Elliott & Smith 2011).	This was to ensure that the child had adequate meta‐cognitive skills to be able to reflect on their own communication through guided video‐based discussion.
Difficulty with conversation as reported by parents and captured in assessment of a videoed conversation. Examples of difficulty include frequent misunderstandings and/or conversations where the ratio of child‐to‐adult speech is markedly low.	DLD is a heterogeneous condition (Bishop et al. 2017). Some children may show relative strengths in conversation, meaning that other areas of language may be a higher priority for intervention.

## 3 Stage 2: Reviewing the Evidence and Selecting Relevant Theory

This stage focused on: (a) conducting a detailed review of existing evidence on interventions to support language and conversation for children and adults with communication disorder and (b) reviewing published research and working with specialist advisers to select relevant theory for the proposed intervention, thereby ensuring that BCDLD has a ‘coherent theoretical basis’ (Craig et al. 2006, p. 4).

### 3.1 Review of the Evidence

#### 3.1.1 Interventions Involving Parents of Children With Language Disorders

Several key studies provide empirical support for training parents of children with language difficulties to use communication support strategies. Roberts and Kaiser (2011) conducted a systematic review and meta‐analysis of parent‐implemented interventions for children aged between 18 and 60 months with primary and secondary language impairments. The results showed large and significant effects for children's expressive language (Hedges *g* = 0.61), compared to untreated controls. Gains for receptive language were moderate and also significant (*g* = 0.35). However, the analysis included studies focusing on children with ASD, Down syndrome and other learning difficulties, as well as DLD. It is also important to note that the Roberts and Kaiser (2011) review includes studies with a high number of intervention sessions (up to 36 h), which may not be realistic within routine clinical practice.

More recently, Roberts et al. (2019) published an updated review, examining the association between parent training and language outcomes for children aged 6 years or younger with, or at risk for, a range of communication impairments. Once again, large and significant effects were reported for children with DLD (*g* = 0.83 for expressive and *g* = 0.92 for receptive language). Children in this diagnostic group also had the strongest social communication outcomes (*g* = 0.37), compared to at‐risk participants, those with ASD or other language and learning impairments.

While an influential study by Boyle et al. (2010) and more recent research by Heidlage et al. (2020) both suggest that difficulties with receptive language may be more difficult to remediate than aspects of expressive language, two scoping reviews by Tarvainen et al. (2020, 2021) conclude that modifying a child's environment (e.g., by training parents and teachers to use facilitative communication strategies) is the most effective form of intervention for both pre‐school and school aged children with language comprehension difficulties.

The results from all of the above studies combine to suggest that interaction‐based therapy has the potential to improve both children's expressive and receptive language, with the latter being identified by Kulkarni et al. (2022) as a priority for language intervention.

#### 3.1.2 Working on Everyday Conversation

There is no available review evaluating the effects of conversation‐based therapy for school‐aged children and very limited literature exists. However, at the other end of the lifespan, there is growing evidence for the use of communication partner training (CPT) to support the everyday conversations of adults with acquired communication difficulties, such as aphasia, and the family, friends and caregivers with whom they interact regularly. Simmons‐Mackie et al. (2010, 2016) conducted two separate systematic reviews, covering a total of 56 studies. Each concluded that CPT is effective in increasing partner skill in facilitating the communication of people with aphasia, however there was insufficient evidence to determine whether this type of intervention is suitable for people with acute aphasia. Furthermore, Cruice et al. (2018) conducted a follow up review of the papers included in the above systematic studies. These authors found that the majority of reported interventions were insufficiently specified to enable replication, while key ingredients, or mechanisms of change, were rarely stated. Furthermore, there was no consensus on optimal dosage or timing of delivery post‐stroke.

The literature on conversation interventions mainly consists of descriptive case studies, or pre‐post designs. Within these studies, a series of papers by Beckley et al. (2013) and Beeke et al. (2011), Beeke et al. (2014), Beeke et al. 2015) provide evidence that significant change in communication behaviours is possible for adults with aphasia and their CPs following an eight‐week manualised programme, ‘Better Conversations with Aphasia’ (BCA). More recently, Volkmer et al. (2023) have reported positive results from an RCT involving an adaptation of BCA for people with primary progressive aphasia. Together, these findings from different populations with acquired communication disorders suggest there is potential for a conversation‐based intervention to be effective for children with DLD and their parents, who are likely to be among their main communication partners.

#### 3.1.3 Strategies Facilitating Language and Conversation

Turning to the specific strategies that are used to promote children's language and conversation within BCDLD, evidence for the effectiveness of recasting, contingent commenting and other facilitative parent/carer behaviours is summarised in Supplementary Materials 2 (e.g., Cleave et al. 2015; Masek et al. 2021; Falkus et al. 2016). There is also evidence that people with aphasia can support, or hinder their own conversations by using specific facilitators, or barrier strategies (see Supplementary Materials 2, e.g., Beeke et al. 2013). These findings underpinned the decision for BCDLD to target both child and parent behaviours within conversation, rather than focusing solely on adult strategies to support their child's language and conversation development.

### 3.2 Selection of Theory to Inform Intervention Development

Though no single published theory acted as a unitary driver for the development of the intervention, the following models of language and social interaction helped guide the design, reflecting the multifactorial nature of communication development and disorders. BCDLD was informed by the social interactionist and constructivist theories of language acquisition. Social interactionists stress the importance of language input to the child, as well as the child's reciprocal uptake of that input. Under this account, children's language development is dependent upon social interaction, which ‘gates’ language learning (Kuhl 2007), such that language ability emerges out of an initial desire to communicate, while the success of this depends on whom we communicate with (Lytle & Kuhl 2017). Building on these ideas, constructivist theories of child language development focus on the collaborative construction of meaning by the child when interacting with adults (Kaufman 2004). Rooted in the work of Vygotsky (1978) and Bruner and Watson (1983), constructivism holds that children's acquisition of new knowledge builds incrementally upon the foundation of previous learning. Thus, adults and children share and acquire language respectively within the ‘zone of proximal development,’ through ongoing problem solving with adult guidance (Vygotsky 1978, p.86). Meanwhile, parents and teachers employ ‘scaffolding’ to continually tailor and adjust the level of support they offer, in response to the child's linguistic level.

Both of the above accounts emphasise the two‐way interaction between children's emerging linguistic skills and parents' ability to scaffold and respond to these, rather than focusing only on the child (nativism) or adult (learnability theory). This dynamic view of language acquisition, for which there is growing evidence (e.g., Romeo et al. 2018a; 2018b) underpinned the decision to focus on both child and adult behaviours as targets for the new intervention.

Insights were also drawn from behaviour change theory (Michie et al. 2009), which focuses on three core components, which can motivate change. These are the *capability (C)* to carry out the desired *behaviour (B)*, including necessary knowledge and skills, the *opportunity* and *motivation (O, M)* to do so (including social, environmental and psychological factors). Johnson et al. (2021, 2017) applied the COM‐B Model of Behaviour (Michie et al. 2011) to investigate mechanisms of change following the ‘Better Conversations with Aphasia’ (BCA) programme. This is a widely‐used framework for understanding and designing behaviour‐change interventions. Seven key mechanisms were identified as activating change in speakers' conversational style following BCA, including increased awareness of their own behaviour (through video reflection) and replacing barriers with facilitators (Johnson et al. 2017). These findings informed the content and focus of the BCDLD programme.

Alongside these theoretical approaches, which are linked to language and behaviour change, BCDLD draws upon systems theory (Hawe et al. 2009), which takes into account the context or setting in which the intervention is delivered and how these interact. For example, BCDLD was designed in accordance with RCSLT clinical guidelines (Taylor‐Goh 2017), including key principles such as: ‘Work in partnership with colleagues both within and outside of the profession in the best interests of service users.’ Parents, as well as children, were considered equal partners in the therapeutic process. Professional conduct and client confidentiality were maintained according to the Health and Care Professions Council standards of proficiency (HCPC). Consideration of children's family history and dynamics also helped tailor several aspects of BCDLD delivery, for example, adapting written materials to support parents who may themselves have a language or learning difficulty and scheduling sessions at the beginning or end of the school day, or during weekends and holidays, to fit around work and childcare commitments.

Finally, BCDLD was influenced by the principles of applied Conversation Analysis (CA), which focuses on how conversational turns are designed and organised within naturally‐occurring talk from the perspectives of the participants themselves. CA considers conversation as collaboratively produced, while language is seen primarily as a tool for social interaction. This viewpoint underpinned the development of BCDLD, which focused on everyday parent‐child interactions as experienced by the child and carer.

## 4 Stage 3 Design of a Logic Model and Therapy Protocol

### 4.1 Development of Logic Model

Programme theory for BCDLD was developed by the project team, with support from the Clinical Advisory Group, as well as experts from UCL's Centre for Behaviour Change. The project team comprised the first author, an experienced SLT who works with school‐aged children with DLD, the second author, an expert in the design of complex interventions in healthcare, the third author, a clinical linguist with specialism in language development and the fourth author, a senior SLT researcher who has expertise in bridging research and practice in this field, and was part of the founding BCA team.

Members of the project and advisory teams reviewed the theories identified above to consider the underlying causal mechanisms which were proposed to drive change in parent‐child conversation. This stage of the process drew on behaviour change theory to identify core principles and characteristics of BCDLD, alongside hypothesised mechanisms of change. The COM‐B framework (Michie et al. 2011) was used to ensure that aspects of capability, opportunity and motivation, as well as socio‐environmental context, were all considered and incorporated into the intervention logic model. This was created to provide a visual representation of programme theory and how the intervention is designed to produce its desired outcomes.

**FIGURE 1:**
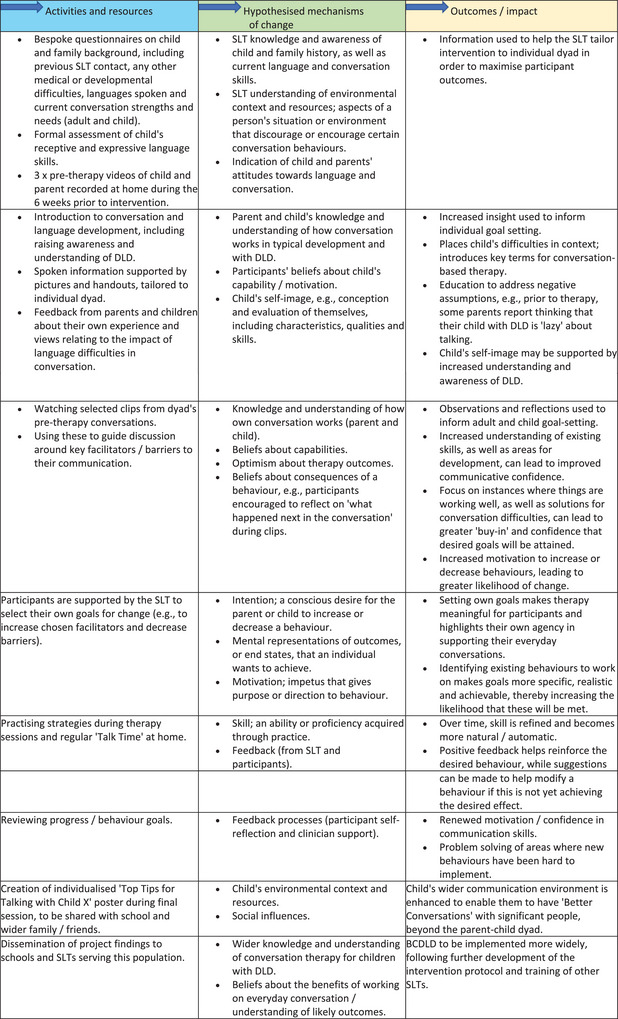
Development of the intervention logic model.

The programme logic model (presented in Figure 1) illustrates how intervention ingredients are hypothesised to lead to changes in child and parent communication behaviours, with outcomes relating to improved insight and understanding of DLD, greater communicative confidence an enhanced communication environment.

Hypothesised mechanisms of change include:

- Awareness‐building through video feedback (e.g., observing the impact of unhelpful behaviours).
- Introduction of alternative strategies (facilitators) through modelling and practice.
- Parent and child co‐learning, enhancing reciprocal responsiveness and adult scaffolding.
- Tailored goals and delivery, aligned to each dyad's individual communication strengths and needs.

### 4.2 Developing the Therapy Protocol

The therapy protocol was developed with reference to the logic model, taking into account the views of parents, children and clinicians to inform aspects of implementation, for example, the content, length and location of sessions. In addition, the first author worked with the Clinical Advisory Group to identify and compare features from existing interventions (e.g., parent‐child interaction therapy and BCA) and to agree which should be adopted or adapted for BCDLD.

The Clinical Advisory Group contributed to decision‐making around the design of the intervention and how to operationalise BCDLD. Core elements were adapted from:

- *Parent–Child Interaction Therapy* (PCIT; Falkus et al. 2016), including focus on language‐enhancing strategies and child‐led communication.
- *Better Conversations with Aphasia* (BCA; Beeke et al. 2013), including use of video feedback and conversation strategy work.

The agreement to include six face‐to‐face sessions was based on the preference of parents and children, with reference to usual clinical practice in the NHS, whereby children on SLT caseloads typically receive a block of 6–8 therapy sessions per term. Separately, it was agreed that BCDLD therapy sessions would be shorter than those typically offered for adult CPT, due to the constraints of school timetabling and limits on children's attention control. A full list of BCDLD components, which were adopted or adapted from existing interventions, is provided in Table 3.

**TABLE 3:** Components from PCIT and BCA which were adopted or adapted for BCDLD.

Therapy component	PCIT	BCA	BCDLD adoption / adaptation
Number of sessions	4	8	6
Length of sessions	Approximately one hours.	1.5 h.	45 min (equivalent to one school period).
Participants	Parents and pre‐schoolers.	Adults with aphasia and CP.	School‐aged children with DLD and their main carers.
Intervention delivered by	Qualified SLT, specialising in Early Years paediatrics.	Qualified SLT, specialising in acquired disorders.	Qualified SLT, specialising in paediatrics.
Use of video	One video recording made in clinic during first PCIT session, to support identification of target strategies. A second video is made in Week 10 (following therapy and consolidation period) to review progress and agree next steps. Further, 5‐min videos are made during therapy sessions to check on progress towards aims.	A total of 18 conversation samples recorded over the course of research therapy (eight pre‐intervention, two during and eight post). All videos were made in participants' homes.	Three pre‐therapy recordings made by participants at home, to support identification of targets. A further home video was made immediately after BCDLD and a final one was recorded at follow up, 6 weeks later. Additionally, participants were encouraged to record further videos during therapy to reflect on progress towards aims. This sometimes included recordings made during therapy sessions.
Interaction context	Play‐based.	Natural conversation.	Natural conversation (but may incorporate games or activities to ‘get the conversation started’).
Therapy aims	To improve the interactions between children with delayed language development and their parents/carers in order to promote child language development.	To change the conversational behaviours of the speaker with aphasia as well as the conversation partner in order to maximise the PWA's turn construction abilities and increase mutual understanding between the dyad.	To adapt the conversation behaviours of both children with DLD and their main carers in order to enhance their everyday conversations and support the child's language development.
Strategies focused upon	Parents only	PWA and CP	Children and parents
Choice of strategies informed by	Child language acquisition evidence and theory.	Conversation analytic studies and knowledge about aphasia and its consequences.	Child language acquisition evidence and theory and CA studies; knowledge about DLD and its consequences.
Selection of strategies	Facilitators only, chosen collaboratively by parent and SLT.	Barriers and facilitators, chosen collaboratively by CP, PWA and SLT.	Barriers and facilitators, chosen collaboratively by parent, child and SLT.
Methods to support strategy choice	Parent and therapist watch pre‐therapy video together. SLT highlights positive strategies used by the parent and introduces a rating scale, highlighting benefits / rationale for each strategy. This scale is used by parents to rate themselves and they are then encouraged to choose a strategy they rated as using ‘never’ or ‘sometimes’ to try using more of at home.	SLT provides information on how conversation works and the impact of aphasia, supported by aphasia‐friendly handouts. He/she then presents short video clips to illustrate patterns within the dyad's own talk. Participants are encouraged to reflect on their own communication and choose from a selection of strategies, suggested by the SLT.	SLT provides information on how conversation works and the impact of DLD, supported by child‐friendly handouts. Participants and SLT repeatedly view clips from pre‐therapy videos and discuss patterns in their talk. Child and parent each select barriers and facilitators to work on, following choices / suggestions from SLT.
Methods used during therapy sessions to support strategy implementation	Regular recordings of parent playing with child during clinic sessions. Parent and therapist watch the video together to see if targets have been achieved / maintained. If so: any changes in the child are highlighted adaptations to the main aim are discussed. If the aim has proved hard to achieve, find out why and then address it together. Revisit why the aim was chosen in the first place.Useful phrases for guiding parent's self‐reflection: What would be the benefit of…? What else could you do? What effect does that have on the child?	Each session introduces a theme to develop participants' understanding of conversation, for example, Session 2 focuses on the concept of repair, using aphasia‐friendly handouts to support discussion of (a) problems in conversation and (b) what happens when things go wrong? (c) three steps involved in repair. Ask dyad to pick things that happen to them. SLT supports this reflection by playing carefully‐selected clips from the dyads' own conversations to illustrate points and reinforce their learning. Practice conversations / role plays during sessions and discussion of these.	Following BCA, each session introduces key information on conversation and language development, supported by child‐friendly handouts or PowerPoint slides. Games and activities are incorporated to maximise children's engagement, for example, playing the ‘Microphone game’ to practise turn‐taking strategies. Video clips made before, or during, therapy are used to illustrate themes relating to the dyad's own communication. Practice conversations and role play are also included to support implementation of strategies, along with discussion / problem‐solving.
Home practice	Parents asked to practise chosen strategies 3–5 times a week during ‘special time’.	Dyad asked to video ‘practice conversations’ at home between sessions.	Parents and children asked to practise strategies 3–5 times a week during ‘Talk Time’.
Reflecting on home practice	Parents asked to comment on their use of ‘special time’ and any changes they have observed in child's communication during subsequent therapy sessions.	Dyad asked to reflect on home strategy use, either by filling in written handouts, or by recording their thoughts.	Dyads asked to complete ‘Talk time record sheet’, detailing their target / activity and comments on how this went, to be discussed during therapy.
Outcome measurement	Parent rating scale, based on first and follow up videos; child mean length of utterance; ratio of child‐to‐adult speech.	Pre‐ and post‐therapy videos analysed to explore changes in: (a) conversation facilitators and (b) conversation barriers.	Pre‐ and post‐therapy videos analysed to explore changes in: (a) conversation facilitators (b) conversation barriers (c) child mean length of utterance and (d) child:adult ratio of speech. In addition, standardised child language measures were administered before and after BCDLD.
Participant views	Published studies on parent and clinicians' views of PCIT, (e.g., Davies et al. 2017; Klatte et al. 2019).	Views of PWA and CPs on BCA intervention, outcome and perceived mechanisms of action (Johnson et al. 2017; 2021)	Views collected from children and parents pre‐ and post‐therapy on children's language, conversation and related skills.

The first author produced a first draft of the intervention protocol, following interest holder input. The Clinical Advisory Group then met to review each aspect of the draft. The views of group members were recorded and, where feasible, amendments were made to reflect their recommendations. Where change was not possible, this was fed back to the group, with an explanation of why these changes were not made. For example, it was suggested that the number of therapy sessions could be reduced from six to four in order to increase treatment efficiency. However, the project team felt this would not allow sufficient time to introduce key information about language and conversation, to identify individual child and parent strategies and allow space for both supported practice and self‐reflection on the impact of changes to communication.

### 4.3 Piloting of the Draft Protocol

Once the draft protocol had been reviewed and refined, this was piloted with two mother‐child dyads, after which their feedback was sought and minor amendments to the protocol were made. Feedback was gathered via questionnaires and informal discussion to gain insight into both children and parents’ experience of participating in the therapy. The first author then met with her Clinical Advisory Group to reflect on barriers and facilitators to therapy delivery. Based on this discussion, together with feedback from the pilot participants, minor pragmatic changes were made to the therapy protocol. For example, Session 5 was modified to include more games and role play activities to help maximise children's engagement. A greater emphasis was placed on using verbal and non‐verbal prompts to encourage the child to use their chosen strategies (e.g., a word or a picture, which was personally selected, to remind them of their target). Finally, the discussion of conversational topic in Session 4 was made more interactive by encouraging dyads to bring in family photographs or favourite magazines as topic starters for practice conversations.

These changes did not affect the underlying theory, structure or focus of the intervention. There was no further change to the content of session plans, therapy materials or delivery context. Table 4 summarises the final agreed theme and content of each session. A full intervention protocol, described using the *TiDier* framework (Hoffmann et al. 2014), is presented in Supplementary Materials 3. This framework is designed to support transparent reporting of intervention components and replicability. Results from an initial evaluation of the programme are published in Hughes et al. (2025).

**TABLE 4:** Summary of intervention sessions.

Session	Theme	Tasks
**1 **	Introduction to conversation and language development	Identify **parent facilitator**; set up ‘Talk Time’ for home practice
**2 **	Turns, sequences and actions	Identify a **child facilitator** to practise at home
**3 **	Trouble and repair	Identify a **parent barrier** / agree an alternative strategy for them to use
**4 **	Child‐led topics of conversation	Use family photos / favourite books as topic starters; practise strategies and identify a **child barrier** behaviour.
**5 **	Consolidation of child strategies	Focus on child strategies, including playing conversation‐based games
**6 **	Reviewing and moving forward	Create a poster for teachers, family and friends to share ‘top tips’ from therapy.

## 5 Discussion

Following a rigorous process informed by O'Cathain et al. (2019) framework for intervention development, we combined interest holder involvement, clinical and academic expertise, with a wide ranging review of relevant practice and theoretical literature to develop the rationale and theoretical basis for a novel intervention for children with DLD, which targets real world language use. In addition, we developed and tested a therapy protocol to deliver the intervention. The next stage of development will be to co‐design a therapy manual and bespoke training programme for practising clinicians.

A strength of the intervention is that it is designed to be implemented flexibly, allowing therapists to tailor the programme to bespoke therapy targets, selected by participants based on their own communication strengths and needs. During the therapy, parents and children view video recordings of themselves in conversation and are supported to reflect on which behaviours support, or hinder, the flow of conversation. Participants are offered a choice of strategies to either practise increasing, or to decrease, often by replacing an unhelpful behaviour, such as parental test questioning, with an alternative, such as commenting, to encourage children's active participation and enjoyment in the interaction. This agency in the process is likely to be a key active ingredient of therapy, though this has not yet been investigated systematically.

Individual adaptations can be made to the delivery of aspects of the intervention, for example, including images and characters that align with the child's personal interests when preparing therapy materials. This corresponds to the recently published Priority Setting Partnership (PSP) Top 10 questions for DLD (RCSLT 2025), which includes the following prioritised questions: ‘How can different people best support people with DLD? How can we best help them to do this?’ And ‘How does help and support need to vary depending on a person's age, background, language abilities and other conditions?’ Across the PSP, there is a consistent call for tailored DLD interventions to be developed, tried and tested, with another of the Top 10 questions asking: ‘What are the best DLD interventions that lead to good and important outcomes that last? Who can best deliver the intervention?’

Many children with DLD have co‐occurring difficulties with auditory memory and / or attention. Additional visual support can be used within BCDLD to help reinforce key concepts and remind children to use their chosen strategies. It may also be appropriate to incorporate movement breaks, additional games and activities in order to promote more sustained participation for children whose difficulties include attention control. For parents and carers with literacy difficulties, written handouts can be replaced with verbal information and/or plain English materials.  The ability to tailor the intervention to the individual dyad is an inherent strength of the intervention (see Figure 1 logic model and Supplementary Materials 3 TiDier description).

### 5.1 Future Research

Phase I development of BCDLD involved extensive consultation with interest holders including parents and children. Wider patient and public involvement (PPI) and co‐production will be key to the next phase of development, including therapy manualisation and wider feasibility testing. Drawing more deeply on the experience of families, alongside best available evidence and clinical expertise, increases the likelihood that the modified intervention will be acceptable and relevant and therefore more likely to result in the desired behaviour change and subsequent communication outcomes.

The next stage of the work is to roll out training and plan further feasibility studies within NHS and education settings to assess intervention acceptability, outcomes, and implementation issues. An important part of this process will be to extend the involvement of parents, young people with DLD, SLTs and school staff to ensure that therapy and recruitment protocols are robust and meet the needs of the widest sample of children with DLD and their main carers.

Uncertainties remain around optimal dosage and session length, long‐term impact and generalisation across settings and wider populations. It is likely that the principles and methods of BCDLD could be adapted for use with children of different ages and with a wider range of developmental disorders, however this remains untested. Extending the application of BCDLD to other clinical groups would require further consultation work with relevant interest holders and a separate evaluation of the adapted intervention.

## 6 Conclusion

This paper has described the Phase I development of a theoretically‐informed, conversation‐based intervention for children with DLD and their conversation partners, which aims to improve the content and flow of their everyday interactions, while supporting children's developing language. Synthesising knowledge from the literature, input from children living with DLD, parents and professionals in the field enabled the design and early‐stage evaluation of this novel intervention, ensuring that BCDLD is ready to progress to Phase 2 feasibility testing.

## Funding

This research was supported by the Economic and Social Research Council, which funded the first author's doctoral training.

## Ethics Statement

The Word Retrieval and Development Project was approved by the University College London Ethics Committee (reference number 281/002).

The Better Conversations with Developmental Language Disorder study was approved by the University College London Ethics Committee (reference number 2981/003).

## Conflicts of Interest

The authors declare no conflicts of interest.

## Supporting information




**Supporting Information**: jlcd70234‐supp‐0001‐SuppMat.docx.

**Supporting Information**: jlcd70234‐supp‐0002‐SuppMat.docx.

**Supporting Information**: jlcd70234‐supp‐0003‐SuppMat.docx.

## Data Availability

The data that support the findings of this study are available on the Open Science Framework at https://osf.io/pwv6f/. Further information about the intervention can be found within the first author's PhD thesis, which is openly available within the following repository: https://discovery.ucl.ac.uk/id/eprint/10187117/.
