# Supplementary material for: ‘Better Conversations With Developmental Language Disorder’: Designing a Novel Intervention for School‐Aged Children and Their Main Carers
Source: Int J Lang Commun Disord. 2026 Apr 15;61:e70234. doi: 10.1111/1460-6984.70234 (PMC13081513; doi:10.1111/1460-6984.70234)
Supplement: Supplementary file 2 — Supporting Information: jlcd70234‐supp‐0002‐SuppMat.docx. [file JLCD-61-0-s002.docx]

### **Supplementary Materials 2:**

### **Summary of evidence for facilitative and barrier communication strategies**

**Parent-child interaction strategies**

1. Recasting/repeating back

Recasts are defined as 'a modified repeat' (Radford et al., 2012), whereby an adult responds to a child's previous utterance by '﻿reflecting it back to them with certain embellishments' (Saxton, 2003). Most commonly, this takes the form of a correction to the child's immature grammar, semantics or phonology, whilst incorporating elements of their original turn and retaining its intended meaning. However, recasts can also entail adult modification of errorless sentences in order to facilitate language acquisition (Clarke et al., 2017). This is known as a 'growth recast' ﻿(Menn, 1993) and can involve replacing or adding new elements to an utterance, for example to introduce a new grammatical construction, which the child has not yet learnt.

Recasting is closely linked to unmodified repetition of what children say, since both provide 'an endorsing receipt' in the next conversational turn (Bruinsma et al., 2020, p. 254). Falkus et al. (2016) note that this has the effect of showing the child that you are listening; confirming their verbal contribution and thereby increasing communicative confidence, while encouraging greater enjoyment of the interaction. In a study of spontaneous conversations between 41 French adult-child dyads, Clark and Bernicot (2008) found that parents sometimes used exact repetitions, combined with rising intonation, to check their understanding. At other times, they followed their repeats with novel information, such that they fulfilled a similar function to recasts. These authors also note that repeats can carry a pragmatic purpose within conversation by helping to establish 'common ground' (Grice, 1981, p.190). This is defined as the 'presumed background information shared by participants' (Stalnaker, 2002, p. 701) and is central to social interactionist theories of language acquisition.

Numerous studies have investigated recasts and repetition in relation to adult input directed at both typically-developing and language-disordered children [(e.g., Camarata et al., 1994; Conti-Ramsden et al., 1995; Fey et al., 1999)](https://theoryandtechniquetool.humanbehaviourchange.org/tool). Together, these studies have shown that parents of both groups naturally recast their children's speech, though this is less frequent for children with DLD than for their TD peers. A recent systematic review by Cleave et al. (2015) found that recasts are an effective technique within grammatical interventions for DLD. However, Fey et al. (2003) argue that language disordered children require recasts at the rate of two per minute in order to benefit from their effects - double the frequency at which they occur during conversation with TD children.

Separately, Radford et al. (2012) provide further evidence that children with DLD may struggle to make use of recasts. They used conversation analysis to examine repair sequences during lessons in a specialist language resource base. Findings show that recasts containing grammatical or phonological corrections were not immediately acknowledged or acted upon by children with language disorder (e.g., through self-repair). Radford and colleagues (2012) suggest that this is due to reduced language processing skills and a tendency to focus on semantic, rather than form-based adult input. These authors recommend a more explicit approach to 'correcting' children's immature output, e.g., by recasting or repeating back an isolated word and by placing tonic stress on the repaired version. However, this runs counter to most SLT approaches, which place emphasis on 'modelling back' mature production without actively exposing the repair or interrupting the flow of conversation.

***2. Contingent commenting***

Contingency is 'the act of responding moment-by-moment to what the child has just done or said' (Bosanquet et al., 2016, p. 46). This adult behaviour is connected to recasting, in that it requires the parent to tune in closely to the child's focus of attention and to respond promptly to their verbal or non-verbal initiations. For example, if the child holds up a toy car, the adult might comment: 'Oh, your toy car!' If the child went on to bang two cars together, the parent could say: 'Oh no! They crashed!' According to Falkus et al. (2016), this style of input benefits children because it gives them access to language which is both accurate and relevant to their interests and experience. Contingent commenting also decreases the pressure on the child to speak, unlike other common parental strategies, such as questioning (considered in Section 2.4.1.4, below).

Studies show that disadvantaged mothers typically engage in less contingent talk with their children than those from higher SES groups (e.g., Hoff, 2003). Meanwhile, infants whose parents frequently use this communication behaviour go on to develop larger vocabularies as toddlers (Masur et al., 2005). A recent randomised control trial (RCT) by McGillion et al. (2017) explored the use of contingent discourse with 142 11-month olds from higher and lower SES families. Caregivers were randomly allocated to either a contingent talk intervention or a control. The intervention was successful in increasing the amount this strategy was used by all caregivers and there was a short-term effect on children’s expressive vocabularies. However, the effects did not maintain for these very young children when participants were followed up at 24 months, indicating that focus on this area alone was not enough to produce lasting benefits.

While contingent commenting is a commonly used technique within PCIT programmes (e.g., Falkus et al., 2016), one potential criticism of promoting this strategy is that it may not be culturally appropriate for all client groups. For example, van Kleeck (1994) points out that while in Western cultures, it is customary for parents to follow a child's lead, this practice may not reflect the underlying values and beliefs of all families participating in SLT intervention. Kwok et al., in preparation, investigated cross-cultural differences in PCI between UK and Hong Kong dyads (N=16; TD children aged between 4 and 8 years, with UK participants drawn from the current study). On average, UK parents used almost three times more contingent comments (*M*=9.75) compared to HK parents (*M*=3.38), while the latter used more teaching behaviours, such as recasts and gave more explanations for words and concepts. Kwok and colleagues suggest that these differences may be linked to underlying cultural beliefs, e.g., Confucian thought (a dominant philosophical value in Chinese society), according to which parents are responsible for educating their child, and value their obedience.

Separately, Burns and Radford (2008) used Conversation Analysis (CA) to examine interactions between three Nigerian mothers and their pre-schoolers. ﻿The study identified a preference for instructional, rather than child-led talk, leading to the recommendation that SLTs should tailor their therapy and advice according to the background and interactional style of each individual dyad. The authors note that mothers who adopt a more directive style of communication, e.g., by giving instructions and explicitly praising or correcting their child, can nevertheless be supported to use more contingent language, e.g., by saying: 'Oh good boy. You broke opened the bricks', rather than simply: 'good boy' (p. 16).

3. Giving clear explanations of words or concepts

Parents often introduce new words to their children within everyday conversation (Clark, 2018). Alongside this, they typically supplement their word offer with information that enables the child to develop their understanding, such as the semantic category, characteristic properties, sounds or actions associated with the target noun or verb. Adults may also contrast the new word's meaning with that of neighbouring items or highlight the relationship between comparable terms (Clark & Wong, 2002). According to Clark (2018), these parental explanations support children to build and organise their own internal semantic representations, which are essential for new word learning. Improving semantic knowledge is also a technique used to remediate word-finding difficulties for children with DLD (Best et al., 2020; Ebbels et al., 2012).

4. Use of questions

One key characteristic of parent-child interactions is the frequency of adult questions directed to the child. While questions can function to establish and maintain young children's attention (Grosse & Tomasello, 2012; Yu et al., 2019) and may encourage children to practise using language (Luo et al., 2022; Mol et al., 2008), research suggests that they can also limit the child's interactional and turn construction opportunities, e.g., by carrying an expectation of a specific response.

Test questions (TQs) account for up to a third of all requests directed at children (Siraj-Blatchford & Manni, 2008). While genuine questions call for the child to provide missing information, TQs solicit knowledge that is 'obviously already known to the questioner'. Also referred to as 'known answer', or 'exam' questions, TQs have been widely studied within educational settings. They typically form part of a three-step question, answer, response (QAR) turn sequence. Under this structure, teachers will initiate a topic by asking a test question (Q) that invites a required answer from students (A), which in turn is given an evaluative adult response (R). This places the child in the position of having to 'display' their knowledge of the correct word or concept - an expectation which is likely to be particularly challenging for children with DLD. Indeed, while questions such as: 'What's that?' were found to contribute positively to receptive language development for typically-developing 2-year-olds (Luo et al., 2022), this association did not hold for children with low vocabularies at this age, suggesting that children with language difficulties may find it challenging to comprehend or respond to these types of referential questions and thus, may miss out on the opportunity to hone their own immature linguistic skills. The Luo et al. (2022) paper also failed to analyse parental follow-up to children's responses, which may have differed as a function of the child's own correct, incorrect or imprecise answer to their originating question.

Another question type, which can be seen as particularly limiting for children's verbal output, is forced choice questions, or 'option-posing prompts' (Brown et al., 2013). These require children to choose between two or more alternatives, e.g.: 'Was it red or white?' or 'Do you want the apple or the orange'? This category of questioning is commonly reported within parent-child interactions (e.g., Cameron-Faulkner et al., 2003; Heather Fritzley et al., 2013; Wells, 1981). Research shows that such closed-ended questions typically elicit shorter and less accurate narratives from children about facts or events they have experienced or witnessed (e.g., Powell et al., 2014). However, younger children and children with DLD may find forced choice questions helpful in some circumstances, as a form of scaffolding which offers them semantically appropriate alternatives which they might otherwise be unable to express. Previous research has shown that it is difficult for children with DLD to respond to more open-ended enquiries (e.g., Brown et al., 2013; Fritzley et al., 2013) because of the higher memory and cognitive load involved in processing these questions.

According to Falkus et al. (2016), reducing the overall use of questions within parent-child interaction can help encourage language development by decreasing the pressure on the child to speak about what the adult already knows. This opens up the child's potential initiation or response opportunities, allowing them to help choose and develop topics that are of interest to them. This, in turn, is likely to elicit more timely and tailored adult feedback, which is essential for ongoing language development (Gillkerson et al., 2017). However, it would not be desirable to eliminate child-directed questions entirely, since these form a fundamental part of natural conversation (Grosse & Tomasello, 2012). Furthermore, as children grow older, they typically use internalised adult models to form their own information-seeking questions, in order to build upon their emerging knowledge and understanding (Clark, 2018).

5. Giving the child extra time to talk.

The final strategy, which is commonly promoted within parent-child interaction therapy, is that of giving the child extra time to speak. The rationale for this is that children with DLD require additional processing time to understand what others have said and to formulate their own contributions to conversation [(Allen & Marshall, 2010; Falkus et al., 2016)](https://www.ncbi.nlm.nih.gov/pmc/articles/PMC6195086/#bib21). Parents may be encouraged to achieve this goal by waiting for the child to start the talking, or by using extended pauses, e.g., leaving a gap of 2 seconds or more within or between speakers' turns (Fox Tree, 2002). According to Falkus et al. (2016), parents' adoption of these facilitative behaviours can help redress the balance of conversation between adults and their children with DLD, whilst also encouraging the child to initiate more.

**Adult conversation strategies:**

1. Example barrier conversation strategies

1.1 Test questions. Just as parents asking children 'known answer' questions can limit interactional opportunities for the younger speaker, so Beeke et al. (2013) have argued that test questions can act as a barrier to the flow of everyday conversation for people with aphasia and may produce negative social and emotional consequences. According to these authors, test questions arise when the CP already knows the answer to their query and is ‘testing’ PWA, either to encourage them to talk, and/or to keep the conversation going. An example TQ would be: 'Tell me the names of your sisters', often followed up by the CP cueing production of a specific name or content word, e.g.: 'One begins with K-'. According to these authors, TQs can cause a 'threat to face' by placing pressure on PWA to produce a specific noun or noun phrase, which may prove elusive despite extensive word search attempts (Burch et al., 2002; Goffman, 1972). Lock et al., (2001) further found that this type of failed word search, prompted by a test question, is often followed by a ‘‘correct production sequence’’; a series of turns designed to prompt the semantic or phonological target, which the PWA is struggling to produce.

In such sequences, CPs may actively withhold information in order to elicit a specific response from the person with communication difficulties, which may not be forthcoming. ﻿Aaltonen & Laakso (2011) have explicitly likened this process to school-based activities, referring to such breakdowns in communication as 'exam halts' (p. 115).

1.2 PWA using minimal or single word turns. Turning to the barrier behaviours identified for PWA participating in the BCA study, Beeke et al. (2013) describe how people with agrammatism frequently use minimal, or single word turns, which highlight their identity as 'a linguistically impaired interactant with minimal resource to take a turn at talk' (p. 799). Within the aphasia literature, a minimal turn is defined as one which:

'does not contribute meaningfully to the conversation, and serves only to hand the conversation back to the other speaker' (Herbert et al., 2013, p. 9).

Typically, minimal turns are composed of tokens, such as 'mmm', 'oh' and 'OK', or a combination of these. While single words differ from minimal turns, in that they contain lexical information, they are grouped together here as they are also a sign of reduced participation by the PWA in conversation. Both minimal and single word turns occur frequently in response to test questions initiated by the non-impaired CP (Beeke et al., 2013).

*1.3 PWA giving up when stuck on a word.* A second PWA barrier, which was identified within the BCA study, involved instances where the PWA abandoned, or failed to complete a turn - often in response to word-finding difficulties. For example, Beckley et al. (2013) described how one participant ('Giles') sought to give up trying to get his message across following an unresolved repair sequence with his wife. The trouble source centred around his difficulty in retrieving a proper noun (Jonathan Ross), which is a common problem within conversation for adults and children with language disorder (Best et al., 2021; Herbert et al., 2003). Prior to therapy, Giles's tendency was to repeat the same indistinct words and gestures multiple times, before surrendering his turn in frustration when his wife could not understand. Instead, he was encouraged to try 'saying it another way' (see Facilitators, below), for example by using a key word, such as 'television' to clearly signal the context for his comments.

While 'giving up when stuck' was identified as a barrier to conversation for this individual dyad, it should be noted that pursuing a specific word across lengthy repair sequences may not always be a desirable strategy. Depending on the context, participants may instead agree to carry on with the conversation to avoid diverting their communicative resources, as in 'correct production sequences' (Lock et al., 2001). Alternatively, these authors suggest that CPs may offer candidate words to try to 'guess' the PWA's meaning. However, this was not a strategy specifically targeted within the BCA study.

2 Example facilitative conversation strategies

2.1 CP uses minimal or passing turns. Whereas using frequent single word and minimal turns was identified as a barrier for PWA participating in the BCA study, this same behaviour was chosen as a target facilitator for several CPs. The rationale for this was that using utterances, such as 'mm hm', 'right', or 'uh huh', rather than adding new information, may prompt the PWA to elaborate or continue with their prior talk (Clark & Schaefer, 1989; Sacks et al., 1974). The advice for CPs to increase their use of minimal (sometimes known as 'passing') turns also appears within the ﻿Supporting Partners of People With Aphasia in Relationships and Conversation programme (SPPARC; Lock et al., 2001), upon which BCA is partly based. According to the SPPARC manual, partners should aim to 'look interested and expectant as you take this type of turn' (p. 32). They are also encouraged to leave an extended pause afterwards, to allow the PWA time to formulate their next utterance or non-verbal contribution.

2.2 PWA uses word finding strategies (e.g., circumlocution, gesture or acting out). These related strategies offer an alternative to the previously discussed barrier of 'giving up when stuck on a word'. Participants within the BCA study were encouraged to use multi-modal turns (e.g., mime, gesture, facial expression and marked prosody) in order to help get their message across within everyday conversation. Where appropriate, some participants also employed drawing and writing as an additional communication resource. Beeke et al. (2013) describe how one PWA, 'Graham' was able to construct turns which, despite his chronic agrammatism, allowed him to convey his opinions and engage actively in conversation - including challenging the views of his unimpaired partner. By using these facilitators, Graham was able to circumvent his significant word-finding difficulties and participate equally with his CP in guiding the topic and flow of the conversation.

It is important to acknowledge that the use of gesture and other physical movement can be problematic for some adults and children with communication disorder - particularly if this co-occurs with dyspraxia (Raymer, 2007; Sinani et al., 2011), as their actions may not be accurate or easily interpretable. However, multiple studies have highlighted the role of non-verbal communication not only in enhancing and extending the information that is being communicated, but also in facilitating lexical retrieval by providing an alternative route to the lexicon. For example, Clough and Duff (2020) highlight the positive effects of gesture on learning and memory for adults with neurogenic conditions, including aphasia. Separately, Goldin-Meadow and Alibali (2013) argue that gesture can support and shape cognition by reducing working memory load for both children and adults with and without communication disorders. When word retrieval fails, multi-modal turns can also be used to help compensate and repair communication breakdowns (Pierce et al., 2019). However, Caute et al. (2021) identify the need for simpler and more reliable tools to code and evaluate gesture, in order to increase understanding of how this can be used facilitatively within SLT intervention.

References:

Aaltonen, T., & Laakso, M. (2011). Halting Aphasic Interaction. Creation of Intersubjectivity and Spousal Relationship in Situ [Article]. *Communication & Medicine*, *7*(2), 95–106. https://doi.org/10.1558/cam.v7i2.95

Beckley, F., Best, W., Johnson, F., Edwards, S., Maxim, J., & Beeke, S. (2013). *Conversation therapy for agrammatism: Exploring the therapeutic process of engagement and learning by a person with aphasia*. 220–239. https://doi.org/10.1111/j.1460-6984.2012.00204.x

Beeke, S., Beckley, F., Best, W., Johnson, F., Edwards, S., & Maxim, J. (2013). Extended turn construction and test question sequences in the conversations of three speakers with agrammatic aphasia. *Clinical Linguistics and Phonetics*, *27*(10–11), 784–804. https://doi.org/10.3109/02699206.2013.808267

Best, W., Hughes, L., Masterson, J., Thomas, M. S. C., Howard, D., Kapikian, A., & Shobbrook, K. (2021). Understanding differing outcomes from semantic and phonological interventions with children with word-finding difficulties: A group and case series study. *Cortex*, *134*, 145–161. https://doi.org/10.1016/j.cortex.2020.09.030

Bosanquet, P., Radford, J., & Webster, R. (2016). *The teaching assistant’s guide to effective interaction: How to maximise your practice* (J. Radford & R. Webster, Eds.) [Book]. New York, N.Y. : Routledge.

Brown, D. A., Lamb, M. E., Lewis, C., Pipe, M.-E., Orbach, Y., & Wolfman, M. (2013). The NICHD Investigative Interview Protocol: An Analogue Study. *Journal of Experimental Psychology. Applied*, *19*(4), 367–382. https://doi.org/10.1037/a0035143

Bruinsma, G., Wijnen, F., & Gerrits, E. (2020). Focused stimulation intervention in 4-and 5-year-old children with developmental language disorder: Exploring implementation in clinical practice. *Language, Speech, and Hearing Services in Schools*, *51*(2), 247–269. https://doi.org/10.1044/2020_LSHSS-19-00069

Burch, K., Wilkinson, R., & Lock, S. (2002). A Single Case Study of Conversation-Focused Therapy for a Couple Where One Partner has Aphasia. *British Aphasiology Society Therapy Symposium Proceedings*, 1–9.

Cameron-Faulkner, T., Lieven, E., & Tomasello, M. (2003). A construction based analysis of child directed speech. *Cognitive Science*, *27*(6), 843–873. https://doi.org/10.1207/s15516709cog2706_2

Caute, A., Dipper, L., & Roper, A. (2021). The City Gesture Checklist: The development of a novel gesture assessment. *International Journal of Language & Communication Disorders*, *56*(1), 20–35. https://doi.org/10.1111/1460-6984.12579

Clark, E. V. (2018). Conversation and Language Acquisition: A Pragmatic Approach. *Language Learning and Development*, *14*(3), 170–185. https://doi.org/10.1080/15475441.2017.1340843

Clark, E. V., & Bernicot, J. (2008). Repetition as ratification: How parents and children place information in common ground. *Journal of Child Language*, *35*(2), 349–371. https://doi.org/10.1017/S0305000907008537

Clark, H. H., & Schaefer, E. F. (1989). Contributing to discourse. *Cognitive Science*, *13*(2), 259–294. https://doi.org/10.1016/0364-0213(89)90008-6

Cleave, P. L., Becker, S. D., Curran, M. K., Owen Van Horne, A. J., & Feyb, M. E. (2015). The efficacy of recasts in language intervention: A systematic review and meta-analysis. *American Journal of Speech-Language Pathology*, *24*(2), 237–255. https://doi.org/10.1044/2015_AJSLP-14-0105

Clough, S., & Duff, M. C. (2020). The Role of Gesture in Communication and Cognition: Implications for Understanding and Treating Neurogenic Communication Disorders. *Frontiers in Human Neuroscience*, *14*, 323–323. https://doi.org/10.3389/fnhum.2020.00323

Fey, M. E., Long, S. H., & Finestack, L. H. (2003). Ten Principles of Grammar Facilitation for Children With Specific Language Impairments. *American Journal of Speech-Language Pathology*, *12*(1), 3–15. https://doi.org/10.1044/1058-0360(2003/048)

Fox Tree, J. E. (2002). Interpreting Pauses and Ums at Turn Exchanges. *Discourse Processes*, *34*(1), 37–55. https://doi.org/10.1207/s15326950dp3401_2

Goffman, E. (1972). *Interaction ritual: Essays on face-to-face behaviour / Erving Goffman* [Book]. Allen Lane.

Goldin-Meadow, S., & Alibali, M. W. (2013). Gesture’s role in speaking, learning, and creating language. *Annual Review of Psychology*, *64*(1), 257–283. https://doi.org/10.1146/annurev-psych-113011-143802

Grice, P. (1981). In *Radical pragmatics / edited by Peter Cole*. Academic Press.

Grosse, G., & Tomasello, M. (2012). Two-year-old children differentiate test questions from genuine questions. *Journal of Child Language*, *39*(1), 192–204. https://doi.org/10.1017/S0305000910000760

Heather Fritzley, V., Lindsay, R. C. L., & Lee, K. (2013). Young Children’s Response Tendencies Toward Yes-No Questions Concerning Actions. *Child Development*, *84*(2), 711–725. https://doi.org/10.1111/cdev.12006

Herbert, R., Best, W., Hickin, J., Howard, D., & Osborne, F. (2003). Combining lexical and interactional approaches to therapy for word finding deficits in aphasia. *Aphasiology*, *17*(12), 1163–1186. https://doi.org/10.1080/02687030344000454

Herbert, Ruth. (2013). *Powers: Profile of word errors and retrieval in speech: An assessment tool for use with people with communication impairment / Ruth Herbert ... [Et al.].* J & R Press.

Heritage, J. (1985). A change-of-state token and aspects of its sequential placement [Bookitem]. In *Structures of Social Action* (pp. 299–345). Cambridge University Press. https://doi.org/10.1017/CBO9780511665868.020

Hoff, E. (2003). The Specificity of Environmental Influence: Socioeconomic Status Affects Early Vocabulary Development via Maternal Speech Author ( s ): Erika Hoff Published by: Wiley on behalf of the Society for Research in Child Development Stable URL : https://www.js. *Child Development*, *74*(5), 1368–1378.

Kleeck, A. van. (1994). Potential Cultural Bias in Training Parents as Conversational Partners With Their Children Who Have Delays in Language Development. *American Journal of Speech-Language Pathology*, *3*(1), 67–78. https://doi.org/10.1044/1058-0360.0301.67

Kwok, Y. T., Hughes, L., Best, W., & Newton, C. (In preparation). *Cross cultural differences in parent-child interaction: Evidence from parents and children from Hong Kong and the United Kingdom.*

Lock, S., Wilkinson, R., Bryan, K., Maxim, J., Edmundson, A., Bruce, C., & Moir, D. (2001). Supporting Partners of People with Aphasia in Relationships and Cconversation (SPPARC) [Article]. *International Journal of Language & Communication Disorders*, *36 Suppl*, 25–30.

Luo, R., Masek, L. R., Alper, R. M., & Hirsh-Pasek, K. (2022). Maternal question use and child language outcomes: The moderating role of children’s vocabulary skills and socioeconomic status. *Early Childhood Research Quarterly*, *59*, 109–120. https://doi.org/10.1016/j.ecresq.2021.11.007

Masur, E. F., Flynn, V., & Eichorst, D. L. (2005). Maternal responsive and directive behaviours and utterances as predictors of children’s lexical development. *Journal of Child Language*, *32*(1), 63–91. https://doi.org/10.1017/S0305000904006634

McGillion, M., Pine, J. M., Herbert, J. S., & Matthews, D. (2017). A randomised controlled trial to test the effect of promoting caregiver contingent talk on language development in infants from diverse socioeconomic status backgrounds. *Journal of Child Psychology and Psychiatry and Allied Disciplines*, *58*(10), 1122–1131. https://doi.org/10.1111/jcpp.12725

Mol, S. E., Bus, A. G., de Jong, M. T., & Smeets, D. J. H. (2008). Added Value of Dialogic Parent-Child Book Readings: A Meta-Analysis. *Early Education and Development*, *19*(1), 7–26. https://doi.org/10.1080/10409280701838603

Pierce, J. E., O’halloran, R., Togher, L., & Rose, M. L. (2019). What Is Meant by ‘Multimodal Therapy’ for Aphasia? *American Journal of Speech-Language Pathology*, *28*(2), 706–716. https://doi.org/10.1044/2018_AJSLP-18-0157

Powell, M. B., Hughes-Scholes, C. H., Smith, R., & Sharman, S. J. (2014). The relationship between investigative interviewing experience and open-ended question usage. *Police Practice & Research*, *15*(4), 283–292. https://doi.org/10.1080/15614263.2012.704170

Radford, J., Ireson, J., & Mahon, M. (2012). The organization of repair in SSLD classroom discourse: How to expose the trouble-source. *Journal of Interactional Research in Communication Disorders*, *3*(2). https://doi.org/10.1558/jircd.v3i2.171

Sacks, H., Schegloff, E., & Jefferson, G. (1974). Linguistic Society of America A Simplest Systematics for the Organization of Turn-Taking for Conversation Author ( s ): Harvey Sacks , Emanuel A. Schegloff and Gail Jefferson. *Linguistic Society of America*, *50*(4), 696–735. https://doi.org/10.2307/412243

Saxton, M. (2003). *Recast in a New Light: Insights for Clinical Practice from Typical Language Studies*. 1–24.

Siraj-Blatchford, I., & Manni, L. (2008). ‘Would you like to tidy up now?’ An analysis of adult questioning in the English Foundation Stage [Article]. *Early Years (London, England)*, *28*(1), 5–22. https://doi.org/10.1080/09575140701842213

Stalnaker, R. (2002). Common ground. *Linguistics and Philosophy*, *25*(5–6), 701–721. https://doi.org/10.1023/a:1020867916902

Wells, Gordon. (1981). *Learning through interaction: The study of language development / Gordon Wells, with contributions by Allayne Bridges ... [Et al.]*. Cambridge University Press.

Yu, Y., Bonawitz, E., & Shafto, P. (2019). Pedagogical Questions in Parent–Child Conversations. *Child Development*, *90*(1), 147–161. https://doi.org/10.1111/cdev.12850
