# Supplementary material for: ‘Better Conversations With Developmental Language Disorder’: Designing a Novel Intervention for School‐Aged Children and Their Main Carers
Source: Int J Lang Commun Disord. 2026 Apr 15;61:e70234. doi: 10.1111/1460-6984.70234 (PMC13081513; doi:10.1111/1460-6984.70234)
Supplement: Supplementary file 3 — Supporting Information: jlcd70234‐supp‐0003‐SuppMat.docx. [file JLCD-61-0-s001.docx]

**Supplementary Materials 3:** Template for Intervention Description and Replication (TIDieR) Checklist for ‘Better Conversations with Developmental Language Disorder’

| Item Number | Item |
| --- | --- |
| 1. | **Brief name**  Better Conversations with Developmental Language Disorder (BCDLD) |
| 2. | **Why**  The aim of BCDLD is to improve communication between children with Developmental Language Disorder (DLD) and their main carers, whilst  promoting the use of supportive communication strategies to  maximise the child’s ongoing language development. This intervention is based on parent-child interaction therapy (Falkus et al., 2016), which is used widely for pre-school children with language needs, and the techniques outlined in the Better Conversations with Aphasia programme (BCA, 2013), which is freely available at: https:// extend.ucl.ac.uk/  BCDLD has been refined to meet the needs of primary school-aged children with DLD and their main adult communication partner (CP). The rationale is that by working with both children and their CP, use of jointly-negotiated communication strategies will be maintained for longer as they can be practised during everyday exchanges at home and target both the child with language disorder and the adult.  The development of BCDLD has been influenced by a) RCSLT Clinical Guidance on the role of Speech and Language Therapists (SLTs) in supporting children with DLD, including working with parents and facilitating communication in functional settings. BCDLD emphasises the joint setting of goals during therapy, facilitated by the SLT, acknowledging key principles from the Special Educational Needs and Disability (SEND) Code of Practice (2014), i.e. that ‘﻿parents know their children best’ (p.79) and that both children and carers must be supported ‘in order to facilitate the development of the child or young person and to help them achieve the best possible educational and other outcomes’ (p.19).  Finally, the intervention is underpinned by behaviour change theory, e.g. therapy aims to: provide multiple opportunities for people to reflect on and practise strategies in therapy sessions and home tasks, using video feedback to support participants to gain awareness of the impact of their communication on one another and focus on elimination of barriers as well as implementation of facilitator strategies. |
| 3. | **What**  Materials:  Each session is accompanied by a session plan, to guide the SLT, outlining specific goals and activities for the session. Participants pre-intervention video-recorded conversation samples will be used to provide clips for video feedback during intervention sessions. These will be filmed on a smartphone or tablet provided by the participant or, where necessary, the Speech and Language Therapist. Handouts and homework sheets have been designed to accompany each session. Additional prompt sheets may be developed with the child and parent during therapy to support them to use specific strategies at home. The following provides a list of handouts and homework sheets used in the sessions:  **Session plan 1: Introduction to Language and Conversation**   - Handout 1: What is conversation about? - Handout 2: What will happen? - Session 1 Homebased task: Introduce regular ‘Talk Time’. - Parent to practise agreed ‘facilitator’ strategy/ies. - Progress to be recorded on ‘Talk Time’ notes sheet.   **Session plan 2: How conversation works (turns, sequences and actions)**   - Handout 3: Turns in conversation - Session 2 Home based task: Child to practise facilitative strategy/ies during ‘Talk Time’. Progress to be recorded on notes sheet.   **Session plan 3: Trouble and repair**   - Handout 4: What can go wrong in conversation / ways to fix it? - Session 3 Homebased task: Parent to practise reducing agreed barrier strategy/ies. Progress to be recorded on notes sheet.   **Session plan 4: Child-led topics of conversation**   - Participants to bring in family photos / favourite books or magazines as topic starters. - Session 4 Homebased task: Child to practise reducing agreed barrier strategy/ies, e.g. by using replacement strategy. Progress to be recorded on notes sheet.   **Session plan 5: Consolidate child strategies**   - Bespoke handouts, according to child’s identified needs and strategies. - Toy microphone to play ‘the microphone game’. - Session 5 Homebased task: Both child and adult to continue practising agreed strategies. Adult to prompt child as necessary to use their chosen facilitators. Progress to be recorded on ‘Talk Time’ notes sheet.   **Session plan 6: Reviewing and moving forward**   - Review videos of early and later sessions to discuss progress towards therapy goals. - Child and carer to produce ‘top tips for conversation’ poster, with SLT support. Print-out to be shared with school and family, as agreed by the participants. |
| 4. | **What**  Procedures: Prior to therapy, the parent and child are asked to record themselves in conversation for at least 5 minutes on three occasions 'talking as they would normally at home'. These videos are then shared with the SLT (e.g. via an encrypted messaging platform, such as WhatsApp or Signal). Recordings are viewed repeatedly by the SLT in order to identify a selection of possible barriers and facilitators to the dyad's conversations. During Session 1, the child and their carer are provided with information and education on conversation (what it is and how it works). The dyad is then supported to reflect on video samples of their own conversation (pre-selected by the SLT) to identify behaviours that facilitate or are a barrier to communication. Subsequently, dyads are facilitated to set goals / identify target communication strategies which they practise during activities, role play and homework tasks. Finally, the dyad is encouraged to share successful communication strategies with others, e.g. teachers, teaching assistants, family and friends. |
| 5. | **Who provides**  BCDLD is designed to be delivered by qualified speech and language therapists who are experienced in working with this population. Ideally, clinicians will have been trained to use parent-child interaction therapy and will have followed the online training modules accompanying the ‘Better Conversations with Aphasia’ intervention program. |
| 6. | **How**  BCDLD is primarily delivered face to face to a child with DLD and their main carer. Both participants are present at all six therapy sessions. Up to two telephone or video conversations may take place in between therapy appointments to answer queries and encourage home practice. |
| 7. | **Where**  Sessions are delivered at home or in a quiet room at the child’s primary school, depending on the availability of parents and the school’s ability to provide a suitable space for therapy to take place. Ideally, at least one session will take place at home to ensure that communication is being worked on in its most natural context. |
| 8. | **When and how much**  The BCDLD program consists of six 45-minute sessions delivered over 6 weeks (half a school term). The following provides an overview of the aims of each intervention session:  **Session 1: Introduction to Language and Conversation**   - Discuss aims of therapy - Discuss and explore what conversation is and what helps - Initial viewing of their own video/s - Parent is supported to identify a ‘facilitator’, i.e. something they are doing well to support their child’s conversation - Introduce ‘Talk Time’ (regular home practice) - Homebased task: Parent to practise increasing use of their agreed ‘facilitator’ strategy/ies - Progress to be recorded by the parent and/or child on ‘Talk Time’ notes sheet   **Session 2: How conversation works (turns, sequences and actions)**   - Review progress from ‘Talk Time’ - Problem-solve any difficulties with setting up practise at home - Discuss how people take turns in conversation (including non-verbal turns) - View videos to explore how parent and child’s turns work - Discuss strategies to help turn-building - Child is supported to select a facilitative conversation strategy/ies - Home based task: Child to practise their chosen strategy/ies during ‘Talk Time’, with parental support. - Progress to be recorded on notes sheet. - Parents encouraged to video at least one ‘Talk Time’ to be reviewed during future therapy sessions.   **Session 3: Trouble and repair**   - Review progress from ‘Talk Time’ - Problem solve any issues that have arisen in using identified strategies in conversations outside of therapy sessions - Discuss what can go wrong in conversation / ways to fix it - View videos to reflect on common breakdowns in conversation and times when the conversation progressed smoothly. - Parent to identify a ‘barrier strategy/ies’, with support from the therapist. - Discuss what they could do differently to help reduce this behaviour. - Home based task: Parent to practise reducing agreed barrier strategy/ies during ‘Talk Time’ - Make new video to be viewed during next therapy session - Progress to be recorded on notes sheet.   **Session 4: Child-led topics of conversation**   - Review progress from ‘Talk Time’ - View videos from home practice sessions to reflect on any positive changes so far. - Child is supported to identify any ‘barrier strategies’ within their own conversation and what they could do differently. - Use family photos / favourite books or magazines as topic starters to practice child strategies in conversation. - Homebased task: Child to practise reducing agreed barrier strategy/ies during ‘Talk Time’, e.g. by using replacement strategy. - Progress to be recorded on notes sheet. - Parents encouraged to video at least one ‘Talk Time’ to be reviewed during therapy   **Session 5: Consolidate child strategies**   - Review progress from ‘Talk Time’ - View videos to reflect on any positive changes from last week. - Bespoke conversation-based games and activities, according to child’s identified needs and strategies, e.g. playing the ‘microphone game’ for turn-taking; using word-webs as prompts for using word-finding strategies or role play to practise asking for help. - Homebased task: Both child and adult to continue practising agreed strategies. Adult to prompt child as necessary to use their chosen facilitators. - Progress to be recorded on ‘Talk Time’ notes sheet. - Parents encouraged to video at least one ‘Talk Time’, if they have not done so previously.   **Session plan 6: Reviewing and moving forward**   - Review progress with ‘Talk Time’ - Watch videos of early and later sessions to discuss progress towards therapy goals. - Problem solve any issues that have arisen in using identified strategies in conversations outside of therapy sessions - Child and carer are supported to produce ‘top tips for conversation’ poster, to be shared with school and family members, as agreed by the participants. |
| 9. | **Tailoring**  Individualised therapy is core to BCDLD. The programme is designed to be tailored for each child with DLD and their main carer / conversation partner) as follows: The approach makes use of video samples of their everyday conversation recorded before and during the intervention. These samples permit the SLT to assess the dyad’s communication strengths and difficulties in order to plan therapy sessions. The use of video feedback also allows participants to reflect on their own conversation style and to set goals i.e. target communication strategies, which they practise during activities, role play and homework tasks. Additional games, activities and visual prompts may be used with children to encourage explicit practice of their identified facilitators within conversation during therapy sessions. These activities and resources are selected according to what motivates the child and what level of support they benefit from to maximise their communicative participation. |
| 10. | **Modifications**  Following consultation with our clinical and wider advisory group, the intervention protocol was modified in order to increase time spent on supporting children to practise their strategies within conversation during therapy, as well as at home. Additional games and role play activities were added to maximise children's engagement (see Session 5). No major changes to the intervention structure, procedures or delivery were made. |
| 11. | **How well**  Planned: This will be assessed in a future study, investigating implementation fidelity for BCDLD when delivered with a wider participant group, administered by trained clinicians working in mainstream schools. Adherence will be assessed using clinician’s notes following each intervention session, as well as a fidelity checklist which will be completed by an independent researcher. The researcher will analyse a random sample of 20% of video recordings of all therapy sessions once the study is completed. Inter-rater reliability will be established by comparing the scores of a second researcher, who will analyse a subset of 20% of the fidelity data. |
